# Time Series miRNA-mRNA integrated analysis reveals critical miRNAs and targets in macrophage polarization

**DOI:** 10.1038/srep37446

**Published:** 2016-12-16

**Authors:** Liangqun Lu, Sara McCurdy, Sijia Huang, Xun Zhu, Karolina Peplowska, Maarit Tiirikainen, William A. Boisvert, Lana X. Garmire

**Affiliations:** 1 Molecular Biosciences and Bioengineering Graduate Program, University of Hawaii at Manoa, Honolulu, HI 96822, USA; 2 Epidemiology Program, University of Hawaii Cancer Center, Honolulu, HI 96813, USA; 3 Genomics Shared Resource, University of Hawaii Cancer Center, Honolulu, HI, 96813, USA; 4 Center for Cardiovascular Research John A. Burns School of Medicine, University of Hawaii Cancer Center, Honolulu, HI 96813, USA

## Abstract

Polarization of macrophages is regulated through complex signaling networks. Correlating miRNA and mRNA expression over time after macrophage polarization has not yet been investigated. We used paired RNA-Seq and miRNA-Seq experiments to measure the mRNA and miRNA expression in bone marrow-derived macrophages over a time-series of 8 hours. Bioinformatics analysis identified 31 differentially expressed miRNAs between M1 and M2 polarized macrophages. The top 4 M1 miRNAs (miR-155-3p, miR-155-5p, miR-147-3p and miR-9-5p) and top 4 M2 miRNAs (miR-27a-5p, let-7c-1-3p, miR-23a-5p and miR-23b-5p) were validated by qPCR. Interestingly, M1 specific miRNAs could be categorized to early- and late-response groups, in which three new miRNAs miR-1931, miR-3473e and miR-5128 were validated as early-response miRNAs. M1 polarization led to the enrichment of genes involved in immune responses and signal transduction, whereas M2 polarization enriched genes involved in cell cycle and metabolic processes. C2H2 zinc-finger family members are key targets of DE miRNAs. The integrative analysis between miRNAs and mRNAs demonstrates the regulations of miRNAs on nearly four thousand differentially expressed genes and most of the biological pathways enriched in macrophage polarization. In summary, this study elucidates the expression profiles of miRNAs and their potential targetomes during macrophage polarization.

Macrophages play important roles in both innate and adaptive immunity. Within various tissue microenvironments, macrophages can adapt and exhibit different phenotypes, and be categorized into two major phenotypes commonly known as M1 and M2. M1 phenotype, referred to as classically activated macrophages (CAM), is an inflammatory state, which produces excessive proinflammatory cytokines and is involved in defense against bacterial, viral and fungal infection. In contrast to the M1 phenotype, M2 macrophages show antagonism to classical activation, down-regulate inflammatory cytokines, and are involved in various functions including tissue repair, homeostasis maintenance and metabolomic processing^1^,^2^. Mantovani *et al*.^3^ proposed that M2 macrophages can be subdivided to three subtypes: M2a (stimulated by IL-4), M2b (stimulated by immune complex + Toll-like receptor ligands) and M2c (stimulated by IL-10 and glucocorticoids). M2a macrophages are known as the classic alternatively activated phenotype and are associated with Th2 responses^4^; M2b macrophages function in a “type 2” immune response, mirroring Th2 T-cell activation and M2c macrophages are featured as deactivated and associated with immunosuppression^4^.

Macrophage polarization is regulated through a complex network including inflammatory modulators, signaling molecules and transcription factors. In M1 macrophages, the signal transduction through JAK1/JAK2 to STAT1/STAT2 initiates the transcription of inflammatory genes involved in M1 polarization, while signaling through JAK1/JAK3 to STAT6 promotes the M2 phenotype^1^,^5^. A series of biomarkers have been identified in the polarization of human and mouse macrophages. In M1 macrophages, inducible nitric oxide synthase (iNOS), which generates nitric oxide from L-arginine, is up-regulated along with the cytokines TNF-α and IL-1β 6. In M2a macrophages, CD206 (mannose receptor C type 1) along with three mouse-specific genes, Ym1, Fizz1 and Arg1 are up-regulated^7^,^8^. However, the regulatory mechanisms that control gene expression involved in phenotypic responses are not fully understood.

Polarized M1 or M2 macrophages behave very differently in various diseases. Tumor-associated macrophages display an M2 phenotype and favor tumor growth and angiogenesis in neoplastic tissues^9^,^10^. Macrophage foam cells induced by western diet (high cholesterol and high fat diet) also present an M2-like anti-inflammatory phenotype^11^, contrary to previous perception that cholesterol overloading would have led to a series of M1 type pro-inflammatory responses^12^,^13^,^14^. This M2-like phenotype is linked to the accumulation of desmosterol, a natural oxysterol and endogenous ligand of Liver X Receptor (LXR) in the LXR pathway, which is well-known for the anti-inflammatory effect and the maintenance of lipid homeostasis in atherosclerosis^15^,^16^,^17^. However, it is observed *in vivo* that a pro-inflammatory response of the typical M1 phenotype is activated, substantially driving plaque development and progression, when macrophages within atherosclerotic lesions encounter modified cholesterol. It remains unclear exactly how the anti-inflammatory response from cholesterol overloading in the plaque macrophages is overwhelmed by the production of complex pro-inflammatory molecules that are typical of M1 phenotype.

To investigate the molecular mechanisms of macrophage polarization, we performed an integrative study of miRNA-mRNA transcriptomic dynamic changes in bone marrow derived macrophages (BMDM) under M1 and M2a conditions. We hypothesize that microRNAs (miRNAs) mediate the early events of polarity switch between M1 and M2a phenotypes, through a complex and dynamic miRNA-targeted mRNA interactome network. MiRNAs are a class of 19–24 nt non-coding RNAs that can regulate genome-wide gene expression by either destabilizing targeted mRNAs and/or inhibiting their protein translation. With advances in identification and functional annotation of miRNAs, it has been established that miRNAs play an important role in regulating immune response, especially in monocytes and macrophages^18^,^19^,^20^,^21^. Although several miRNA studies have been previously published investigating M1 and/or M2 macrophages^5^,^22^,^23^, these studies either only provided a “snap-shot” picture of miRNA profile at a single time point, or lacked the systematic integration of the miRNA-mRNA interactome, leading to limited discoveries.

To overcome these limitations, we designed our study using a time-series (1, 2, 4 and 8 hrs) to examine the expression profiles of paired miRNAs and mRNAs in polarized primary mouse bone marrow derived macrophages (BMDM), using next-generation sequencing platforms. Our aim was to identify: (1) the miRNAs that are robustly and differentially expressed (DE) between M1 and M2 macrophages; (2) the genes and biological functions that are targeted by DE miRNAs; and (3) whether miRNA-mRNA targetome interactions mediate the polarization of macrophage phenotypes. Our results provide detailed identification of miRNA-target regulatory networks during the early stage of macrophage polarization, which may aid in the development of future therapeutic applications for diseases involving macrophage activation.

## Results

### Summary of experimental design and quality assessment

**Figure 1:**
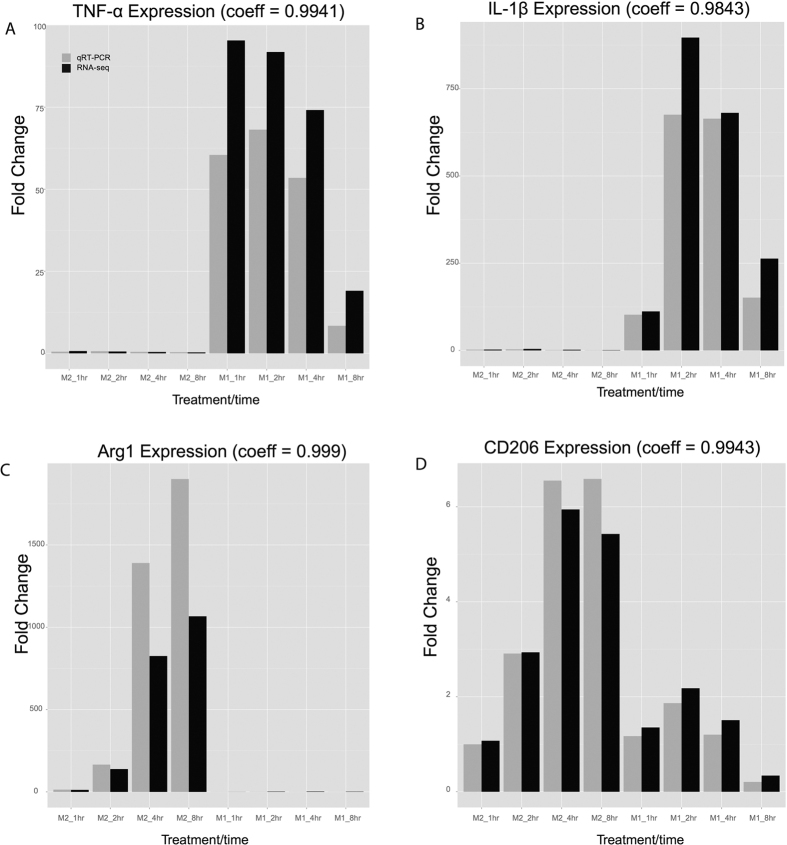
Validation of gene expression of four markers for macrophage polarization. Bar plot of marker gene expression fold change at 4 time points in M1 and M2 macrophages, based on qRT-PCR (gray) and or averaged RNA-seq (black) results. **A–D**: TNF-α, IL-1β, Arg1 and CD206 expression, respectively.

To reveal the transcriptome of polarized macrophages, we sequenced paired mRNAs and small RNAs from M1 macrophages (induced by IFNγ at 10 ng/mL and LPS at 50 ng/mL) and M2a macrophages (induced by IL-4 at 10 ng/mL) at four time points (1, 2, 4 and 8 hours), with duplicates at each time point. Each duplicate was pooled from three C57BL/6 wild type mice. To ensure that the proper phenotypes were induced, we conducted qRT-PCR experiments and confirmed the expression levels of two M1 marker genes TNF-α and IL-1β as well as two M2 marker genes Arg1 and CD206 (Fig. 1).

### Expression profiling analysis of miRNA-seq data

To analyze miRNA profiles in M1 and M2 macrophages, we conducted standard adapter removal and aligned the reads to mouse miRNA annotation file downloaded from miRbase (version 21)^24^, using miRDeep2^25^ which enabled the discovery of novel miRNAs. We obtained an average of 7.8 million filtered reads per sample, with the average mapping rate of 77.6% (Table S1). Next we performed differential expression (DE) analysis of miRNAs between M1 and M2 macrophages, using two criteria (1) an average fold change between M1 vs. M2 over all time points greater than 1.5; and (2) statistical significance with “two-factor” (M1/M2 condition and time) matrix design using R package limma^26^, where the pairing information is considered among samples extracted at the same time.

**Figure 2:**
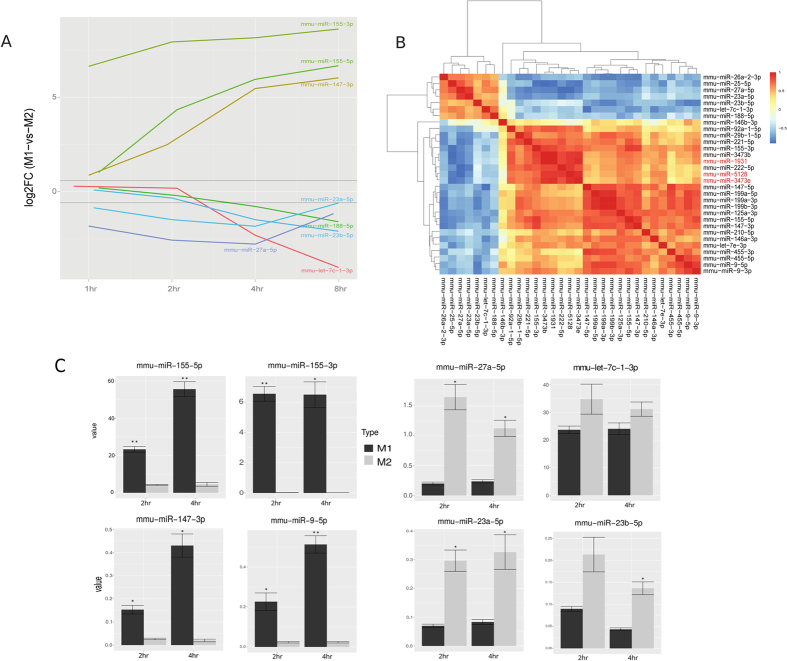
Differentially expressed (DE) miRNAs in mouse M1 vs. M2 macrophages. (**A**) log2 averaged fold-change of differentially expressed miRNAs at 4 time points, with highlights on a few miRNAs with the most drastic changes over time. (**B**) Hierarchical clustering among DE miRNAs based on Spearman’s correlation coefficient (SCC). The three less-studied miRNAs are highlighted by red. (**C**) qPCR validation of top 4 differentially expressed miRNAs for M1 and M2 conditions respectively, at 2 and 4 hours. (n = 3, *p < 0.05, **p < 0.001).

As a result, we obtained 31 robust DE miRNAs between M1 and M2 macrophages over the 4 time points, 13 of which had higher than 1.5 fold changes at any time point (Table S2). As shown in Fig. 2A, of the 24 miRNAs that were expressed consistently higher in M1 macrophages, miR-125a-5p, miR-29b, miR-155 (miR-155-3p and miR-155-5p) and miR-145-5p were previously identified to be expressed abundantly in M1 macrophages^22^,^23^. Interestingly, three miRNAs which have not been assigned to any miRNA family, miR-1931, miR-3473e, and miR-5128, also had high levels of expression in M1 macrophages (Fig. 2B and Table S2). On the other hand, 7 miRNAs including miR-27a-5p, miR-23a-5p, miR-23b-5p, let-7c-1-3p and miR-188 were expressed at significantly higher levels in M2 macrophages. Among them, miR-26a and let-7c were previously reported to be more abundant in M2 macrophages^22^,^27^. To validate the miRNA-Seq results, we repeated the cell culture experiments for 2 hours and 4 hours under the same conditions, and conducted qPCR on the top 4 miRNAs specific for M1 and M2 conditions, respectively. The trends of changes in these miRNAs obtained from qPCR correspond well with those in miRNA-Seq (Fig. 2c). Specifically, miR-155-3p, miR-155-5p, miR-147-3p and miR-9-5p, all have significantly higher expression levels in M1 cells, whereas miR-27a-5p, let-7c-1-3p, miR-23a-5p and miR-23b-5p, all have higher expression levels in M2 cells.

To identify relationships between these 31 signature miRNAs, we calculated the correlations amongst them over the time course (Fig. 2B). Besides anti-correlations of M1 and M2 signature miRNAs as expected, we also discovered that M1 signature miRNAs can be divided into two sub-clusters. Sub-cluster 1 includes miR-3473b, miR-222-5p and miR-29b-1-5p, as well as the newer miRNAs miR-1931, miR-3473e, and miR-5128, all of which are tightly correlated. On the other hand, sub-cluster 2 is composed of the previously reported M1-specific miRNAs and/or their complements, such as miR-155-3p, miR-9-5p/3p, miR-147-5p/3p, miR-125a-3p and let-7e-3p. Such expression trends show that sub-clusters 1 and 2 can be classified as early- and late-response miRNAs, respectively (Fig. S1, Table S3). Early- vs. late-response transcripts in M1 macrophages have been identified for mRNAs but not miRNAs^28^. Here, using a time-series design we were able to tease out these two types of miRNAs as well.

### Expression profiling analysis of mRNA-Seqdata

**Figure 3:**
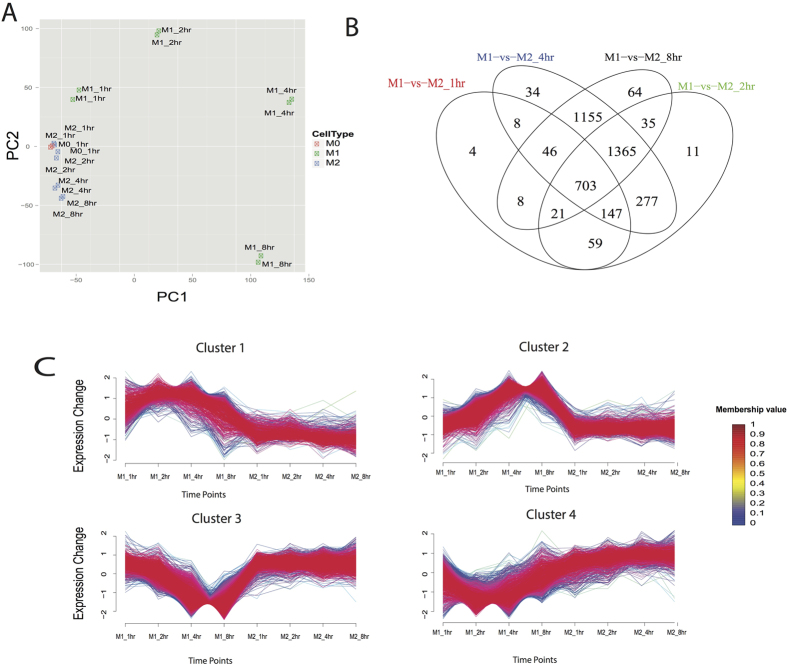
Differentially expressed (DE) genes in mouse M1 vs. M2 macrophages. (**A**) Principal Component Analysis (PCA) plot of 18 samples based on all detected genes in RNA-seq (**B**) Venn diagram of DE genes at 4 time points. (**C**) The time-series trends of 4 clusters of DE genes, where each line represents the fold change relative to M0 state (inactivated macrophage at 0 hr) of one gene. Red color indicates stronger membership and green/blue color denotes weaker membership, as seen in the legend. Heatmap plot based on membership values was seen in Fig. S2. The enriched pathways in each cluster can be seen in Table S7.

To analyze mRNA profiles in M1 and M2 macrophages, we obtained an average of 30.8 million reads per sample after quality filtering and mapping rate of 96.6% (Table S4). To assess the quality of mRNA-Seq data globally, we performed PCA analysis of mRNA transcriptome in all M0, M1 and M2 samples (Fig. 3A). Consistent with earlier observations^29^, M2 is located closer to M0 on the 2D PCA plot, whereas M1 cells deviate from M0 dramatically even after 1 hour of activation (Fig. 3A). Moreover, the fold changes of specific M1 or M2 marker genes between mRNA-Seq and qRT-PCR measurements are highly correlated (correlation coefficients of 0.984–0.999). The M1 marker genes TNF-a and IL-1β and the M2 marker genes Arg1 and CD206 were used (Fig. 1), to confirm the high quality of the mRNA-Seq data.

Next we performed DE analysis on mRNA transcriptomes between M1 and M2 macrophages, similar to that for miRNAs. We identified 3937 genes as DE genes (Tables S4 and S5) over the time-course, which represents a wide dynamic range of changes over time. At 1 h, a small number of these DE genes have a fold change over 2, while numerous other genes are differentially expressed at later time points (Fig. 3B and C).

To investigate the functional dynamic changes of DE genes, we conducted clustering analysis according to similar expression pattern using R package Mfuzz^30^, and then performed KEGG pathway analysis on each cluster. Considering expression patterns and biological functions, we separated the DE genes into 4 optimal clusters (Fig. 3C, Fig. S2 and Table S7). The genes in Clusters 1 and Cluster 2 have higher expressions in M1, compared to M2 macrophages. Cluster 1 has a decreasing trend of gene expression, with enriched pathways in signal transduction and immune response. Cluster 2 has an increasing trend and is also enriched with pathways for immune response and antigen processing and presentation. Although Clusters 1 and 2 have similar functions, their expression trends show that they are composed of early- and late-response genes, respectively. For example, the Jak-STAT signaling pathway is enriched both in Cluster 1 and 2, but it participates in both early- and late-responses by utilizing different subsets of genes. Pik3cb, Pik3r5 and interferon family members Ifna2, Ifnb1 and Ifna4 are early-response genes found in Cluster 1, while the Stat family members Stat1-Stat5, Jak2, Jak3, Akt2, Akt3, Pik3cb and Pik3cg are late-response genes found in Cluster 2.

Conversely, genes in Cluster 3 and Cluster 4 have higher expression in M2 compared to M1 polarized macrophages. In particular, Cluster 3 has high and stable expression in M2 macrophages, and is enriched in pathways involved in cell growth, DNA replication and pyrimidine metabolism. As such, it is postulated to be involved in cell proliferation and immune regulation^31^,^32^. On the other hand, Cluster 4 has increasing expression levels in M2 macrophages over time, and is enriched in the genes Cry2, Per1, Per2, Per3 and Nr1d1, which are involved in circadian rhythm. In summary, we have found significant differences of gene expression profiles between M1 and M2 macrophages. M1 is characterized by immune responses and signal transductions, whereas M2 is involved in basic survival mechanisms, such as cell growth and proliferation, and metabolic processes.

### Functional analysis of pathways and leading edge genes targeted by DE miRNAs

To identify the targetome of DE miRNAs, we intersected the predicted miRNA target results of targetScan with the mRNAs which have negative correlations (Spearman’s correlation coefficients < −0.5) with the miRNAs. As a result, we obtained 4464 putative targets for 31 DE miRNAs. To search for evidence of experimental validations on the 31 DE miRNAs, we checked them in two relatively comprehensive databases miRTarbase^33^ and miRWalk^34^ to search for evidence of experimental validation. Six miRNAs had experimentally validated targets from miRTarbase^33^ and ten others had recorded targets from miRWalk^34^ (Table S9). Among them, miR-9-5p and miR-155-5p were the miRNAs with the highest number (over 20) of experimentally validated targets. Fisher’s exact tests on the overlapped targets between experimentally based databases and our computational prediction yielded p-values of 1.12e-38 and 3.45e-05 for miR-9-5p and miR-155-5p, respectively, indicating that our integration method yielded biological relevance.

**Figure 4:**
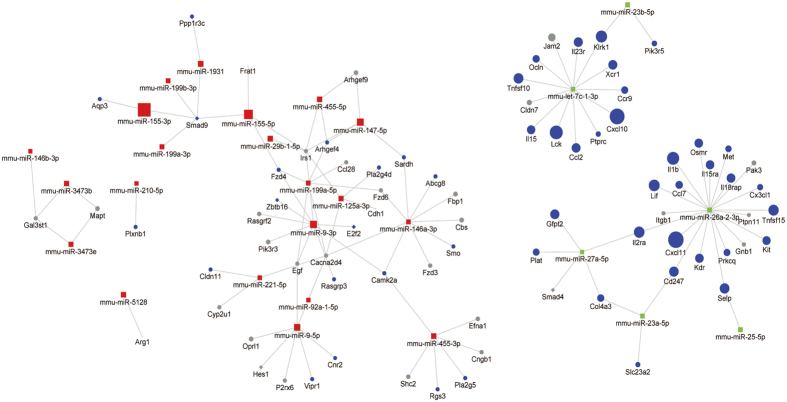
The network of DE miRNAs and leading genes targeted by each DE miRNA. Gene Set Enrichment Analysis (GSEA) was performed on the predicted targets of each miRNA, in order to obtain the leading genes of the significant (P < 0.05) pathways targeted by that particular miRNA. The color labels are: red (M1-specific miRNAs), green (M2-specific miRNAs), blue (DE and leading edge genes) and gray (non-DE and leading edge genes). The node size is proportional to the average fold change of M1 vs. M2 macrophages. The node shapes of diamond and circle denote transcriptional factors (TFs) and other non-TFs genes, respectively.

To systematically reveal the biological processes that each miRNA is related to, we conducted Gene Set Enrichment Analysis (GSEA) on predicted targets per miRNA. We list the top ranked pathways according to the P-values (Table S10), and demonstrated the network of miRNAs and their leading edge genes of significantly targeted pathways (P < 0.05) in Fig. 4. Many miRNAs act on the same biological pathways. For example, miR-199a-5p, let-7c-1-3p and miR-26a-2-3p all target Cytokine-cytokine receptor interaction, with P-value less than 0.03. The synergy at the pathway level is largely attributed from the molecular level, where many miRNAs have common leading edge gene targets. For example, miR-155-5p, miR-155-3p, miR-199a-3p, miR-199b-3p and miR-1931 all target SMAD9. Smad9 is one of the R-smads that transduce the signals initiated from bone morphogenetic proteins in the TGF-beta (Transforming growth factor beta) pathway. The fact that multiple M1 specific miRNAs target SMAD9 may provide a molecular mechanism as to how the TGF-beta pathway is repressed in M1-polarized macrophages.

On the other hand, the M2-specific miRNAs have two dominant clusters of leading edge gene targets by miR-26a-3p and let-7c-1-3p. Although only let-7c-1-3p has been reported to be an M2-specific miRNA, our data indicate miR-26a-2-3p has even more targets than let-7c-1-3p. Interestingly, most of the targets of these two miRNAs are immune response genes. Let-7c-1-3p targets Ccr9, Il15, Cxcl10, Ccl2 and Lck, whereas miR-26a-2-3p targets Il15ra, Ccl7, Cx3cl1, Cxcl11, Il1b and Prkcq. Additionally, miR-27a-5p, miR-26a-3p and miR-23a-5p form a closed loop of association, mediated through IL2ra (targeted by miR-27a-5p and miR-26a-2-3p), Cd247 (targeted by miR-26a-2-3p and miR-23a-5p) and Col4a3 (targeted by miR-23a-5p and miR-27a-5p). IL2ra and Cd247 are cell surface receptors, encoding interleukin-2 receptor alpha and T-cell receptor zeta respectively. IL2ra is involved in PI3K-Akt signaling pathway and Jak-STAT signaling pathway, whereas Cd247 is a component of the T-cell receptor-Cd3 complex, which functions in coupling antigen recognition and intracellular signaling. Our results indicate that miR-26a-2-3p, miR-27a-5p and miR-26a-2-3p may attenuate inflammatory response in M2 macrophages by down-regulating IL2ra and Cd247. Additionally, Col4a3 encodes Type IV collagen alpha 3 and participates in PI3K-Akt signaling pathway. Deficiency of Col4a3 was reported to be associated with non-activated macrophages^35^, consistent with our observation that it is repressed in the M2 phenotype.

### Network analysis of DE miRNAs and their targeted DE mRNAs

**Figure 5:**
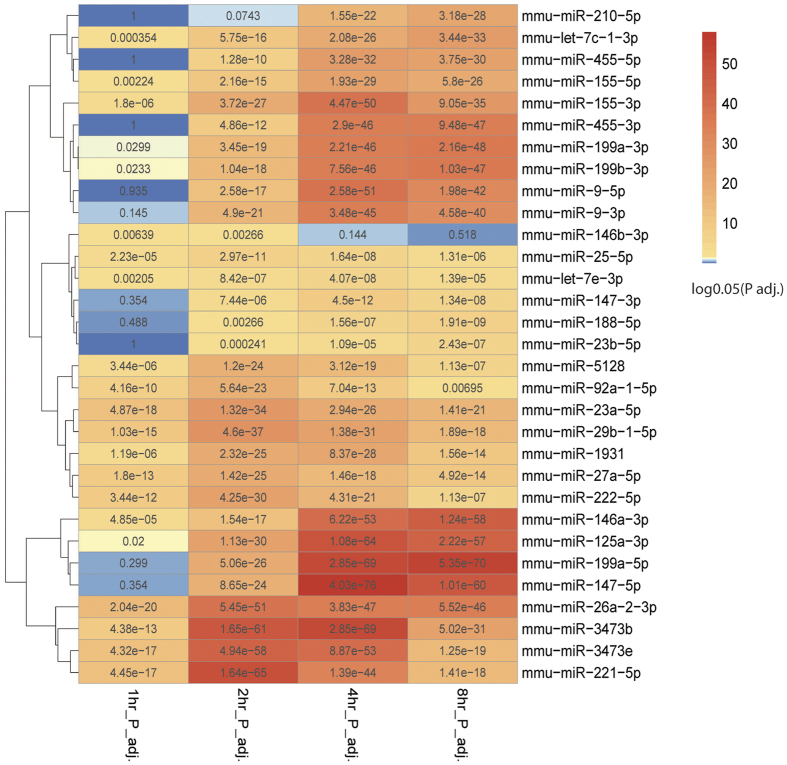
Heatmap to show the global enrichment of miRNA targets among DE genes. For each time point, hypergeometric tests were performed to verify if the miRNA targets are indeed enriched among the DE genes, and the Benjamin-Hochberg(BH) adjusted p-value is then log(0.05) transformed per miRNA per time-point. Red indicates statistical significance, and blue indicates none. The miRNAs are clustered to demonstrate their relationships.

Here we focused on direct and integrative targetome analysis of both DE miRNAs and targeted DE mRNAs, rather than the list of top pathways and leading edge genes targeted by individual miRNAs, as done in the previous section. Among the 3937 DE genes, 2095 (53.2%) are predicted targets of the 31 DE miRNAs. To quantitatively assess the effects of miRNAs on gene DE over time, we conducted hypergeometric tests. The results show that miRNA target genes are highly enriched among DE genes, as 29 out of 31 DE miRNAs have significantly enriched target genes (adjusted P-value < 0.05) in at least three out of four time points (Fig. 5). Moreover, among DE genes of Clusters 1 to 4, the miRNA targets account for 40.4%, 40.4%, 62.7% and 64.9% respectively, suggesting that miRNAs may play even more important roles in M2 phenotype induction compared to that of M1. Almost all 38 significant pathways in each cluster of DE genes comprise genes of miRNAs targets, except antigen processing and presentation in Cluster 2 (Table 1). The largest percentage of miRNA targets comes from the p53 signaling pathway, where 11 out of 13 DE genes are targets of 19 miRNAs. These results provide strong evidence that miRNAs broadly and dominantly affect many of the biological processes associated with M1 and M2 macrophages.

**Figure 6:**
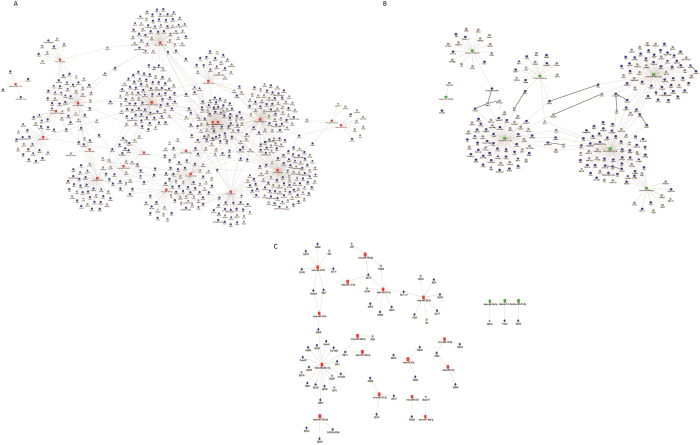
Highly-correlated M1- and M2-specific miRNA-target network. The miRNAs with higher expression levels in M1 (**A**) and M2 (**B**) macrophages, are shown with their highly correlated targets (SCC > 0.8) that are predicted by targetScan. (**A**) Highly correlated miRNAs and targets in M1 macrophages. (**B**) Highly correlated miRNAs and targets in M2 macrophages. (**C**) Highly correlated miRNAs and C2H2 zinc-finger families member targets in both M1 and M2 macrophages. The node shape and color labels are the same as Fig. 4.

Since the relationship between miRs and targets are complex, with one miRNA potentially targeting multiple genes and multiple miRNAs targeting one common gene, we next conducted network analysis to illustrate their complex interactions. The network consists of 10264 pairs of DE miRNAs and target genes, among which 1095 pairs (including 29 miRNAs and 864 gene targets) have more than 0.8 negative correlation that we used for further analysis. The network of miRNAs enriched in M1-polarized macrophages is composed of 22 miRNAs and 634 genes. Of these target genes, 419 are down-regulated significantly in M1-polarized macrophages, as seen in Fig. 6A. The two hub genes targeted by the highest numbers of miRNAs are Eif2c4 and Tmem106b. Eif2c4 is presumably a target of 5 miRNAs including miR-9-5p and miR-199a-5p/3p. It encodes protein AGO4, a component of RISC that binds to short RNAs such as microRNAs (miRNAs) and represses the translation of mRNAs^36^,^37^. Consistent with this result, RISC was reported repressed early on during inflammation as a response to the translation of the proinflammatory cytokines during macrophage activation^38^. Tmem106b encodes Transmembrane Protein 106B, regulating lysosomal morphology and function. Previous research has shown that Tmem106a, another member of the same family, promoted an M1 phenotype through the activation of MAPK and NF-κB pathways^39^. Additionally, among the DE targets in this highly correlated miRNA-target network, 5 immune response genes, 7 cell growth genes and 19 metabolic genes were identified. Their average fold changes in M1- versus M2-polarized macrophages is shown in Figs S3–S5.

The network of miRNAs enriched in M2-polarized macrophages is composed of 7 miRNAs and 230 target genes, of which 143 are down-regulated significantly in M2-polarized macrophages (Fig. 6B). Interestingly, Cflar, Ptpn2 and Tnfsf15 are common targets of miR-27a-5p and miR-26a-2-3p; Pvr, Arid5a, Pik3r5 and Hbegf are common targets of miR-27a-5p and miR-23a-5p (in addition to Col4a3 which was identified earlier in the GSEA analysis), and Gpd2 is a common target of let-7c-1-3p and miR-25-5p. Consistent with the repression patterns in M2 in our study, these genes were largely shown to be expressed in M1-polarized macrophages. Pik3r5 (Phosphoinositide-3-Kinase Regulatory Subunit 5) and Ptpn2 (Protein Tyrosine Phosphatase Non-Receptor Type 2) are phosphatases repressed in M2. Their expression may promote M1 macrophages, since macrophage activation and polarization rely on phosphorylating and activating signaling proteins during the signaling transduction processes^40^. Other genes, such as Tnfsf15 (Tumor Necrosis Factor member 15), Hbegf (Heparin-Binding EGF-Like Growth Factor), and Cflar (CASP8 And FADD-Like Apoptosis Regulator) mainly located in the plasma membrane are involved in NF-κB and MAPK signaling pathways, known to be active in M1 macrophages. Additionally, Pvr (poliovirus receptor) and Arid5a (AT Rich Interactive Domain 5 A) are known to be induced by LPS^41^,^42^. Gpd2 (Glycerol-3-Phosphate Dehydrogenase 2) is prevalent in mitochondria and possibly activates NF-κB and MAPK by producing ROS^43^. Besides the hub genes, 12 immune response genes are identified to be repressed in M2 (Fig. S3). Overall, our results reveal that miR-27a-5p, miR-26a-2-3p, let-7c-1-3p, miR-25-5p and miR-23a-5p boost M2 phenotype through repressing multiple genes related to NF-κB and MAPK signaling pathways.

Collectively, our network analysis shows that correlating with miRNA regulation, the majority of immune targets have higher expression in M1 macrophages while miRNA targets involved in cell growth and metabolism are active in M2 macrophages. Among the miRNA targets, transcription factors account for over 10% (92) of the targets, of which the vast majority (84) are in the M1-network. Surprisingly, about 67% (56) of the targeted transcription factors are within the C2H2 zinc-finger family, with 44 of them differentially expressed between M1 and M2-polarized macrophages (Fig. 6C). Most C2H2 zinc-finger genes are repressed in M1 macrophages, with the exception of Hivep2, Zfp456 and Zfp516.

### Functional validation of novel miRNAs in macrophage polarization

**Figure 7:**
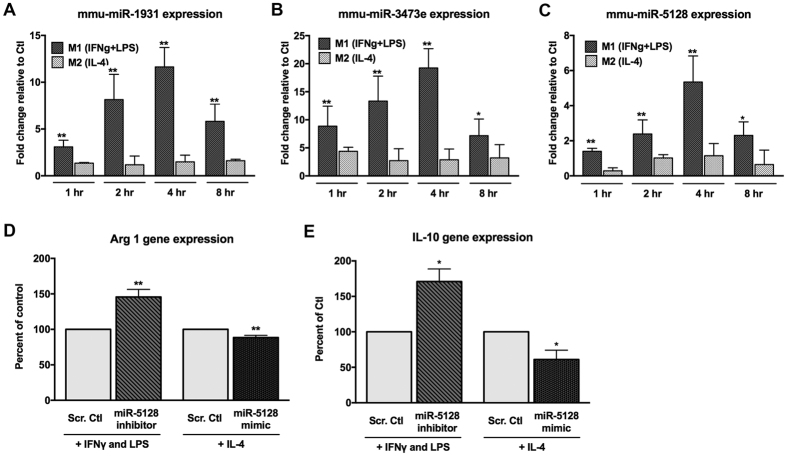
Validation of 3 new miRNAs in macrophage polarization. (**A–C**) qRT-PCR validation for the expression of the three miRNAs miR-1931 (**A**), miR-3473e (**B**), miR-5128 (**C**), using primary BMDM polarized to the M1- or M2- phenotypes after 1, 2, 4, or 8 hours. (**D,E**) BMDM transfected with miRCURY LNA microRNA inhibitors or mimics of miR-5128 (100 μM) for 24 hours followed by M1 polarization for 2 hours or M2 polarization for 4 hours were analyzed by qRT-PCR for M2-associated gene expression. Changes in Arginase 1 (Arg1) (**D**) and Interleukin-10 (IL-10) (**E**) transcript levels are displayed as a percentage of M1- or M2-polarized, scrambled control-transfected cells. (n = 3, *p < 0.05, **p < 0.001).

Since three new miRs (miR-1931, miR-3473e, and miR-5128) upregulated in M1-polarized macrophages (Fig. 2C and Table S2) do not yet have a miRNA family designation, we decided to conduct a functional validation experiment of these miRs using qRT-PCR (Fig. 7A–C). RNA transcripts of miR-1931, miR-3473e, and miR-5128 from primary BMDM cells after M1 polarization are significantly more abundant than those in M2 condition. Moreover, their expression values in M1 all decrease similarly after 4 hr, confirming that they are M1 early response miRs. In order to test the biological function, we chose miR-5128 for primary BMDM transfection with its inhibitors or mimics, along with a corresponding scrambled inhibitor or mimic control. After 24 hours, the mimic-transfected cells were treated with IL-4 for 4 hours to induce M2-polarized gene expression, while the inhibitor-transfected cells were treated with IFNγ + LPS to induce M1 gene expression. The expression of Arg1 transcript, a predicted target of miR-5128 (Fig. 4), was up-regulated by the miR-5128 inhibitor and down-regulated by the miR-5128 mimic (Fig. 7D), indicating that miR-5128 indeed directly targets Arg1. Additionally, miR-5128 inhibition led to the upregulation of Interleukin-10, a potent anti-inflammatory cytokine, while the mimic significantly suppressed IL-10 gene expression (Fig. 7E). Given the known role of Arg1, and the important anti-inflammatory function of IL-10 in M2 polarization, miR-5128 may prove to be an important target of interest for modulating macrophage inflammatory phenotypes.

## Discussion

The aim of this study was to fine map the expression of miRNAs in macrophage polarization, and provide the first interactome of miRNAs and their targetome. To achieve this, we sequenced paired mRNAs and miRNAs from primary mouse bone marrow derived M1- and M2-polarized macrophages with up to 8 hours of induced polarization. We identified 31 robust DE miRNAs that provide signatures of M1 and M2-polarized macrophages. These miRNAs exhibited substantial regulation during macrophage polarization, correlating with a majority of the nearly four thousand DE genes identified, as well as nearly all of the biological pathways analyzed.

Our time series analysis has revealed that not only mRNAs, but also miRNAs, can be categorized into early- and late-response elements. The early- and late-response miRNAs present in M1 macrophages exhibit strong positive correlations between members within the same group. On the other hand, early- and late-response mRNAs are present in both M1- and M2-polarized macrophages. During M1 polarization, the early- and late-response DE mRNAs are involved in immune response/signal transduction through different functional gene subsets. In M2 macrophages, early-response DE mRNAs are involved in cell growth/metabolism, while the late-response DE mRNAs have enriched function relating to circadian rhythm. M1 macrophages are involved in proinflammatory activation while M2 macrophages engage in cell proliferation and metabolism.

This study has confirmed the regulation of some previously observed miRNAs during macrophage polarization, shown in Table S12^22^,^23^,^44^. For example, we have confirmed high expression of miR-155, miR-9, miR-146a and miR-19 in M1-polarized macrophages^22^,^44^,^45^. We have also confirmed that miR-26a-2-3p and let-7c are expressed at higher levels in M2-polarized macrophages, as described previously^23^,^27^. Additionally, we have identified three less-studied miRNAs, miR-5128, miR-3473e and miR-1931, as M1-specific early-response miRNAs.

We also observed several discrepancies between our study and others. For example, miR-27a-5p was reported higher in M1-polarized macrophages by Graff JW *et al*.^22^, however our study shows that miR-27a-5p is significantly up-regulated in M2 macrophages. The differences could be due to several reasons, such as the time and dosage difference of the treatment. Although both studies used identical conditions to induce an M1 phenotype, we used a lower dose of IL-4 at 10 ng/mL, compared to their 20 ng/mL to induce the M2a phenotype. Also, we profiled the gene expression in earlier time points (up to 8 hrs), while Graff JW *et al*. investigated the prolonged treatment effect after 72 hrs^22^. Thus it is possible that there is an initial surge of miR-27a-5p M2 but then its expression is higher in M1 after prolonged treatment.

To thoroughly investigate the functions of miRNAs, we utilized two in-depth complementary network analysis approaches. In the first approach, we investigated the targetome of each miRNA in parallel using the GSEA analysis of the predicted targets, and obtained the top pathways and their leading edge genes targeted by each miRNA. The resulting network of miRNA-leading edge genes can be regarded as a sub-network with the most important functional genes. It is through this approach that we identified that a hub gene SMAD9, was repressed in M1 macrophages by multiple miRNAs. In the second approach, we explored more comprehensive DE miRNA - targetsome interactions using the combined filtering of targetScan prediction and correlation threshold (cc > 0.8). The majority of immune-related genes, along with the phosphatase genes Pik3r5 and Ptpn2, and the NF-κB signaling related genes Tnfsf15, Hbegf, Cflar and Gpd2, have high expression in M1 macrophages compared to M2 macrophages, which have high expression of genes related to cell growth and metabolism.

One of the most interesting findings of this study is that a relatively large number of C2H2 zinc-finger genes among the transcription factors targeted by miRNAs, largely due to sequence similarities in their 3′UTR regions. The function of these zinc-finger proteins in DNA binding and immune response regulation^46^,^47^, were previously reported to be post-transcriptionally regulated by miR-23, miR-181, miR-188, and miR-199 through seed-match in conserved coding regions^48^,^49^. Here we have found that they are targeted by 29 out of 31 DE miRNAs. The functions of C2H2 family are diverse, including both transcriptional activators and suppressors, although suppressor functions are more often found^50^. The majority of the C2H2 genes were shown to be repressed following LPS stimulation^51^, consistent with our results. Future research may elucidate the exact mechanism of miRNAs regulation of the C2H2 zinc-finger family.

In order to validate the 3 newly identified M1-early response miRNAs (miR-1931, miR-3473e, and miR-5128) and test their functional relevance in macrophage polarization, we performed experiments using mimics and inhibitors of each miR in primary BMDM. Our discovery that Arg1 is a bona fide target of miR-5128 implicates that miR-5128 inhibition may be beneficial in treating pathologies involving macrophage inflammation. While Arg1 is a predicted direct target of miR-5128 according to Targetscan^52^, IL-10 does not show complementary binding sites for miR-5128. Thus, it is likely that IL-10 is an indirect target of miR-5128. Although further investigation is needed to more thoroughly identify the regulatory mechanism of the 3 new miRNAs on target genes, our findings set the groundwork for further exploration of the biological functions of novel miRNAs in macrophage polarization. Based on our findings, regulation of miR-5128 appears to be a promising measure for inducing an anti-inflammatory macrophage phenotype.

Other important points of discussion concern the complex spectrums of M1 and M2 macrophage phenotypes. We have followed the experimental conditions widely accepted in the field of macrophage biology and well documented by others^53^, with IFNγ and LPS for M1 polarization and IL-4 for M2a phenotype induction. IFNγ and LPS may induce slightly different but largely overlapping changes in gene expression in M1^29^. One obvious caveat to our study is that the *in vitro* induction of macrophage polarization cannot be extrapolated to *in vivo* conditions. Despite the limitation of our work (similar to others published within the field), we believe that our data and bioinformatics analysis are the most complete thus far in terms of the involvement of miRNAs and their targets in macrophage polarization.

In summary, we have deciphered functional miRNAs in the polarization of macrophages through the repressed targets, including immune response genes, NF-κB signaling genes and phosphatase genes as well as cell cycle and metabolic genes. Additionally, our research has shown that C2H2 zinc-finger family members are the majority of targeted transcriptional factors by DE miRNAs.

## Materials and Methods

The authors confirm that all methods were carried out in accordance with relevant guidelines and regulations, and that all experimental protocols were approved by University of Hawaii’s Institutional Biosafety Committee.

### M1 and M2 primary macrophage cell culture and RNA extraction

Bone marrow cells were extracted from both femurs and tibias of 6–8 week old male C57/BL6J mice. The isolated cells were strained through a 40 μm cell strainer, then plated at 25 × 10^6^ cells per 15-cm plate for macrophage differentiation in DMEM supplemented with 10% FBS, 20% L929-conditioned media and 1% penicillin/streptomycin. The use of L-929 conditioned medium as a source of M-CSF in primary murine BMDM culture has been standard protocol and has been described in detail previously^53^. Adherent macrophages were replated on day 7 into 6-well plates at 3 × 10^6^ cells per well in medium without L929 conditioned media supplementation. After overnight incubation, the macrophages were treated with 1) IFNγ (10 ng/mL) + LPS (50 ng/mL) or 2) IL-4 (10 ng/mL) in duplicate for 1 hr, 2 hr, 4 hr or 8 hr, in order to induce M1 or M2 phenotype, similar to previous published studies^22^,^29^. Untreated macrophages in duplicate were used as controls and collected at the 1hr time point. Cells were collected and centrifuged at 400 × G for 5 minutes. Cell pellets were used for total RNA extraction with an miRNeasy mini kit (Qiagen), designed to purify genomic total RNAs including mature miRNAs. The quality and quantity of the nucleotide samples were assessed using the Agilent Bioanalyzer and Nanodrop technologies (University of Hawaii Genomics Shared Resources Facility).

### Quantitative Real-Time PCR (qRT-PCR) validation of marker genes

In order to verify the expression data generated by mRNA-seq, we performed qRT-PCR experiments for polarization marker genes. Total RNA was isolated with TRIzol (Invitrogen). cDNA was synthesized from 1 μg of total RNA in a 20-μl reaction with qScript cDNA Synthesis kit (Quanta Biosciences). qRT-PCR for the following genes: TNFα, IL-1β, Arg1 and CD206 was performed for each sample in triplicate and Gapdh served as internal control. qRT-PCR was performed using the 2x FastStart Universal SYBR Green Master Mix (Roche) and primers were purchased from Integrated DNA Technologies (IDT). Samples were run on an Applied Biosystems 7900HT Fast Real-Time PCR System.

### Read mapping

Small RNA sequencing and mRNA sequencing were done in Yale Keck Genomic Cores, both at 75 bp read-length with single-end reads. For RNA-seq data, raw reads were removed adapters and aligned to UCSC mouse mm10 genome using Tophat (v2.0.8b)^54^. Default parameters were used (2 base mismatches allowed in the read, and 0 allowed in the splice regions). HTSeq^55^ was used to calculate the raw count numbers based on the mm10 gff file. For miRNA-seq data, raw reads were calculated by miRDeep2^25^ by mapping to mouse miRNA annotation file (with 1280 miRNAs) downloaded from miRbase (version 21)^24^.

### Differential expression analysis

The raw counts of mRNA- and miRNA-seq were normalized between samples using DEseq. A total of 16531 genes were detected in M1 or M2 macrophage. In miRNA annotation file, A total of 790 miRNAs were detected in M1 or M2 macrophage, among which 507 miRNAs had maximum count numbers over 10 reads after normalization. The 16531 genes and 507 miRNAs were subject to differential expression analysis using the R package limma^26^, with two-factor design to consider both time and sample conditions as two factors. For RNA-seq data, genes were considered DE when the averaged absolute fold-changes at four time points were greater than 2 and the adjusted p-values were less than 0.05. For miRNA-seq data, miRNAs were considered DE when the averaged absolute fold-change at four time points were greater than 1.5 and the adjusted combined p-values less than 0.05.

### Time series clustering of differentially expressed genes

The R package Mfuzz (v2.30)^30^ was used to cluster DE genes along time series. Mfuzz is a soft-clustering method based on fuzzy c-means algorithm. Average expression values at each time point were log2 transformed as the input to generate clusters based on the expression trend. The genes were assigned to each cluster with membership values over 0.5 and 98.86% (3892/3937) belonging to a unique cluster.

### MiRNA target prediction

MiRNA target prediction was based on Targetscan^52^. Mouse UTRs were downloaded from the targetscan website, and mature sequences of miRNAs were downloaded from miRBase^24^. Pre-computed miRNA targets were downloaded from the targetscan website. For the miRNAs that were not in the pre-computed summary file, the targets were predicted using a perl script on the Targetscan website. Additionally, we used the correlation criteria to refine the predicted miRNA targets. we calculated the spearman correlation coefficient (SCC) in each miRNA-target pair across all samples, and chose miRNA-target pairs predicted by targetScan with SCC < −0.5 and adjusted P-values of SCC statistical test <0.05.

### Network and function enrichment analysis

DAVID (v6.7)^56^ was used for KEGG analysis on DE genes, with Benjamini-Hochberg adjustment for p-values. Gene Set Enrichment Analysis (GSEA)^57^ was used to obtain significantly enriched pathways and the leading genes targeted by each miRNA. Cytoscape 3.0^58^ was used to display the miRNA-target network. The protein-protein interaction downloaded from NIA Mouse Protein-Protein Interaction Database^59^ was used to infer the network among targets, with specification of mouse TF data downloaded from AnimalTFDB^60^. High-degree targets were obtained through network analysis in Cytoscape.

### Transfection with microRNA inhibitors and mimics for functional validation

Primary mouse bone marrow derived macrophages were isolated and differentiated as described above and 3 × 106 cells per well of a 6-well plate were seeded for experiments overnight in DMEM supplemented with 10% FBS and 1% pen/strep. miRCURY LNA^TM^ miRNA inhibitors or mimics for mmu-miR-1931, mmu-miR-3473e, or mmu-miR-5128 were purchased from Exiqon. X-tremeGENE siRNA transfection reagent (Roche) was used to transfect the cultured macrophages with the miRNA mimics or inhibitors at a final concentration of 100 nM in 2 mL of fresh media for 24 hours. Following transfection, the cells were washed once with PBS and miRNA inhibitor-transfected cells were treated with IFNγ (10 ng/mL) + LPS (50 ng/mL) for 2 hours, while miRNA mimic-transfected cells were treated with IL-4 (10 ng/mL) for 4 hours.

### Quantitative Real-Time PCR (qRT-PCR) validation of miRNAs

RNA was extracted as described above. cDNA was synthesized from the miRNA inhibitor and mimic-treated samples, as well as the original polarized samples (M1 and M2; 1 hr, 2 hr, 4 hr, and 8 hr) using the miScript II RT kit (Qiagen). qRT-PCR was performed using miScript Primer Assays for mmu-miR-1931, mmu-miR-3473e, or mmu-miR-5128, mmu-miR-155-3p, mmu-miR-155-5p, mmu-miR-147-3p, mmu-miR-9-5p, mmu-miR-27a-5p, mmu-let-7c-1-3p, mmu-miR-23a-5p and mmu-miR-23b-5p, as well as RNU6 (control). The miScript SYBR Green PCR Kit (all Qiagen) was used to detect the levels of each miRNA.

## Additional Information

**How to cite this article**: Lu, L. *et al*. Time Series miRNA-mRNA integrated analysis reveals critical miRNAs and targets in macrophage polarization. *Sci. Rep.* **6**, 37446; doi: 10.1038/srep37446 (2016).

**Publisher's note:** Springer Nature remains neutral with regard to jurisdictional claims in published maps and institutional affiliations.

## Supplementary Material

Supplementary Information

Supplementary Tables

## Figures and Tables

**Table 1:** The enriched pathways and potentially regulatory DE miRNAs.

Cluster	Term	p-value	BH-adjusted P	DE_miRNAs
c1	mmu04060:Cytokine-cytokine receptor interaction	2.21E-23	2.65E-21	mmu-miR-188-5p,mmu-miR-23a-5p,mmu-miR-26a-2-3p,mmu-miR-27a-5p,mmu-let-7c-1-3p,mmu-miR-25-5p
c1	mmu04620:Toll-like receptor signaling pathway	2.11E-11	1.27E-09	mmu-miR-23a-5p,mmu-let-7c-1-3p,mmu-miR-26a-2-3p,mmu-miR-23b-5p,mmu-miR-27a-5p
c1	mmu04621:NOD-like receptor signaling pathway	1.23E-09	4.91E-08	mmu-let-7c-1-3p,mmu-miR-26a-2-3p,mmu-miR-23a-5p
c1	mmu04210:Apoptosis	5.23E-09	1.57E-07	mmu-miR-23a-5p,mmu-let-7c-1-3p,mmu-miR-26a-2-3p,mmu-miR-27a-5p,mmu-miR-23b-5p
c1	mmu04630:Jak-STAT signaling pathway	2.18E-08	5.22E-07	mmu-miR-23a-5p,mmu-miR-25-5p,mmu-miR-27a-5p,mmu-let-7c-1-3p,mmu-miR-23b-5p,mmu-miR-26a-2-3p
c1	mmu04623:Cytosolic DNA-sensing pathway	1.44E-07	2.88E-06	mmu-let-7c-1-3p,mmu-miR-26a-2-3p,mmu-miR-27a-5p
c1	mmu04622:RIG-I-like receptor signaling pathway	1.17E-05	2.01E-04	mmu-miR-188-5p,mmu-let-7c-1-3p,mmu-miR-23a-5p,mmu-miR-27a-5p,mmu-miR-23b-5p,mmu-miR-26a-2-3p
c1	mmu04010:MAPK signaling pathway	1.44E-05	2.17E-04	mmu-miR-26a-2-3p,mmu-miR-23a-5p,mmu-let-7c-1-3p,mmu-miR-27a-5p
c1	mmu04062:Chemokine signaling pathway	9.62E-05	1.28E-03	mmu-miR-23a-5p,mmu-let-7c-1-3p,mmu-miR-23b-5p,mmu-miR-26a-2-3p,mmu-miR-27a-5p
c1	mmu04660:T cell receptor signaling pathway	2.16E-04	2.59E-03	mmu-miR-23a-5p,mmu-let-7c-1-3p,mmu-miR-27a-5p,mmu-miR-23b-5p,mmu-miR-26a-2-3p
c1	mmu04640:Hematopoietic cell lineage	4.39E-04	4.77E-03	mmu-miR-188-5p,mmu-miR-23a-5p,mmu-miR-26a-2-3p,mmu-miR-23b-5p,mmu-miR-27a-5p,mmu-miR-25-5p
c1	mmu04115:p53 signaling pathway	1.49E-03	1.48E-02	mmu-miR-188-5p,mmu-miR-27a-5p,mmu-let-7c-1-3p,mmu-miR-26a-2-3p
c2	mmu04062:Chemokine signaling pathway	3.36E-07	5.18E-05	mmu-miR-188-5p,mmu-miR-23a-5p,mmu-miR-27a-5p,mmu-miR-25-5p,mmu-miR-26a-2-3p,mmu-let-7c-1-3p
c2	mmu04630:Jak-STAT signaling pathway	1.23E-06	9.49E-05	mmu-miR-188-5p,mmu-miR-27a-5p,mmu-miR-25-5p,mmu-let-7c-1-3p,mmu-miR-26a-2-3p,mmu-miR-23b-5p,mmu-miR-23a-5p
c2	mmu04620:Toll-like receptor signaling pathway	2.32E-06	1.19E-04	mmu-miR-23a-5p,mmu-miR-26a-2-3p,mmu-let-7c-1-3p
c2	mmu04622:RIG-I-like receptor signaling pathway	4.59E-06	1.77E-04	mmu-miR-23a-5p,mmu-let-7c-1-3p,mmu-miR-26a-2-3p,mmu-miR-27a-5p
c2	mmu04623:Cytosolic DNA-sensing pathway	4.70E-05	1.45E-03	mmu-miR-23a-5p,mmu-miR-26a-2-3p,mmu-miR-23b-5p,mmu-miR-27a-5p
c2	mmu04510:Focal adhesion	8.29E-04	1.41E-02	mmu-miR-188-5p,mmu-miR-27a-5p,mmu-miR-25-5p,mmu-miR-26a-2-3p,mmu-miR-23b-5p,mmu-let-7c-1-3p,mmu-miR-23a-5p
c2	mmu04621:NOD-like receptor signaling pathway	6.60E-04	1.44E-02	mmu-miR-188-5p,mmu-miR-23b-5p,mmu-let-7c-1-3p,mmu-miR-26a-2-3p
c2	mmu05416:Viral myocarditis	8.11E-04	1.55E-02	mmu-miR-188-5p,mmu-let-7c-1-3p,mmu-miR-23a-5p,mmu-miR-26a-2-3p
c2	mmu04060:Cytokine-cytokine receptor interaction	6.41E-04	1.63E-02	mmu-miR-23a-5p,mmu-let-7c-1-3p,mmu-miR-27a-5p,mmu-miR-25-5p,mmu-miR-26a-2-3p,mmu-miR-23b-5p
c2	mmu05221:Acute myeloid leukemia	1.26E-03	1.93E-02	mmu-miR-188-5p,mmu-miR-27a-5p,mmu-miR-25-5p,mmu-miR-26a-2-3p,mmu-let-7c-1-3p
c2	mmu05211:Renal cell carcinoma	1.87E-03	2.58E-02	mmu-let-7c-1-3p,mmu-miR-26a-2-3p
c2	mmu04514:Cell adhesion molecules (CAMs)	2.80E-03	3.27E-02	mmu-miR-25-5p,mmu-miR-26a-2-3p,mmu-let-7c-1-3p
c2	mmu04940:Type I diabetes mellitus	2.75E-03	3.48E-02	mmu-miR-26a-2-3p
c2	mmu04210:Apoptosis	3.65E-03	3.68E-02	mmu-miR-188-5p,mmu-miR-23a-5p,mmu-miR-26a-2-3p,mmu-let-7c-1-3p,mmu-miR-27a-5p
c2	mmu04666:Fc gamma R-mediated phagocytosis	3.57E-03	3.86E-02	mmu-miR-188-5p,mmu-miR-25-5p,mmu-miR-23b-5p,mmu-let-7c-1-3p,mmu-miR-26a-2-3p
c2	mmu04662:B cell receptor signaling pathway	5.42E-03	4.81E-02	mmu-miR-188-5p,mmu-miR-26a-2-3p,mmu-let-7c-1-3p
c2	mmu04612:Antigen processing and presentation	5.29E-03	4.98E-02	
c3	mmu04110:Cell cycle	5.10E-12	8.73E-10	mmu-miR-199a-5p,mmu-miR-199a-3p,mmu-miR-3473b,mmu-miR-125a-3p,mmu-miR-455-5p,mmu-miR-221-5p,mmu-miR-199b-3p,mmu-miR-155-5p,mmu-let-7e-3p,mmu-miR-210-5p,mmu-miR-147-5p,mmu-miR-9-3p,mmu-miR-9-5p,mmu-miR-146a-3p,mmu-miR-455-3p,mmu-miR-155-3p
c3	mmu03030:DNA replication	6.94E-07	5.93E-05	mmu-miR-3473b,mmu-miR-155-5p,mmu-miR-147-5p,mmu-miR-9-5p,mmu-miR-3473e,mmu-miR-155-3p
c3	mmu03440:Homologous recombination	2.32E-05	1.32E-03	mmu-miR-199a-3p,mmu-miR-147-5p,mmu-miR-9-3p,mmu-miR-199b-3p
c3	mmu03430:Mismatch repair	3.26E-05	1.39E-03	mmu-miR-199a-5p,mmu-miR-3473b,mmu-miR-125a-3p,mmu-miR-147-5p,mmu-miR-3473e,mmu-miR-155-3p,mmu-miR-9-5p
c3	mmu00240:Pyrimidine metabolism	2.68E-04	7.60E-03	mmu-miR-125a-3p,mmu-miR-199a-5p,mmu-miR-199a-3p,mmu-miR-3473b,mmu-miR-1931,mmu-miR-155-3p,mmu-miR-210-5p,mmu-miR-3473e,mmu-miR-221-5p,mmu-miR-199b-3p,mmu-miR-9-5p,mmu-miR-455-3p,mmu-miR-222-5p,mmu-miR-455-5p,mmu-miR-146a-3p,mmu-miR-147-5p,mmu-miR-155-5p,mmu-miR-29b-1-5p
c3	mmu04114:Oocyte meiosis	2.54E-04	8.65E-03	mmu-miR-199a-3p,mmu-miR-125a-3p,mmu-miR-199a-5p,mmu-miR-3473b,mmu-miR-29b-1-5p,mmu-miR-146a-3p,mmu-miR-147-5p,mmu-miR-9-3p,mmu-miR-210-5p,mmu-let-7e-3p,mmu-miR-9-5p,mmu-miR-155-3p,mmu-miR-455-5p,mmu-miR-199b-3p,mmu-miR-155-5p,mmu-miR-455-3p,mmu-miR-3473e
c3	mmu04914:Progesterone-mediated oocyte maturation	7.25E-04	1.76E-02	mmu-miR-199a-3p,mmu-miR-125a-3p,mmu-miR-199a-5p,mmu-miR-3473b,mmu-miR-9-3p,mmu-miR-210-5p,mmu-let-7e-3p,mmu-miR-146a-3p,mmu-miR-155-3p,mmu-miR-455-5p,mmu-miR-199b-3p,mmu-miR-155-5p,mmu-miR-147-5p,mmu-miR-9-5p,mmu-miR-455-3p
c3	mmu04115:p53 signaling pathway	1.05E-03	2.22E-02	mmu-miR-125a-3p,mmu-miR-199a-3p,mmu-miR-199a-5p,mmu-miR-5128,mmu-miR-1931,mmu-miR-3473b,mmu-miR-199b-3p,mmu-let-7e-3p,mmu-miR-221-5p,mmu-miR-9-5p,mmu-miR-210-5p,mmu-miR-155-3p,mmu-miR-455-5p,mmu-miR-147-5p,mmu-miR-9-3p,mmu-miR-146a-3p,mmu-miR-155-5p,mmu-miR-455-3p,mmu-miR-222-5p
c4	mmu04710:Circadian rhythm	3.18E-04	4.23E-02	mmu-miR-3473b,mmu-miR-199a-5p,mmu-miR-125a-3p,mmu-miR-1931,mmu-miR-155-3p,mmu-miR-3473e,mmu-miR-221-5p,mmu-miR-92a-1-5p,mmu-miR-9-3p,mmu-miR-222-5p,mmu-miR-210-5p,mmu-miR-147-5p,mmu-miR-9-5p,mmu-miR-146a-3p
