# Supplementary material for: Time Series miRNA-mRNA integrated analysis reveals critical miRNAs and targets in macrophage polarization
Source: Sci Rep. 2016 Dec 16;6:37446. doi: 10.1038/srep37446 (PMC5159803; doi:10.1038/srep37446)
Supplement: Supplementary Information [file srep37446-s1.pdf]

Time Series miRNA-mRNA integrated analysis reveals critical miRNAs and targets in macrophage polarization

Liangqun Lu[1,2*], Sara McCurdy[3*], Sijia Huang[1,2], Xun Zhu[1,2], Karolina Peplowska[3], Maarit Tiirikainen[3], William A. Boisvert[4§], Lana X Garmire[1,2§]

Supporting Information

Figure S1 Expression patterns of early-response and late-response DE miRNAs. For the 24 DE miRNAs highly expressed in M1 macrophages, early-response and late response DE miRNAs are in two sub-clusters. The expression patterns of these sub-clusters are shown in M1_1 and M1_2.

Figure S2. Time series clustering membership heatmap. The membership values of each gene to each cluster are shown in the heatmap.

Figure S3. Highly correlated miRNA-target network--immune response genes. 19 immune related genes and the associated miRNAs were obtained from the highly correlated network, and the interaction was shown in the heatmap. The average fold change of M1 versus M2 macrophages for each gene was filled in the cell when the interaction existed.

Figure S4. Highly correlated miRNA-target network--cell growth genes. 7 cell growth related genes and the associated miRNAs were obtained from the highly correlated network, and the interaction was shown in the heatmap. Similar to Figure S3, the average M1/M2 fold change was filled when the interaction existed.

Figure S5. Highly correlated miRNA-target network--metabolic genes. 19 metabolic genes and the associated miRNAs were obtained from the network, and the interaction was shown in

the heatmap. Similar to Figure S3, the average M1/M2 fold change was filled when the interaction existed.

Supplementary Tables

Supplementary Table 1. Mapping rates in small RNA sequencing from 18 macrophage samples.

Supplementary Table 2. The fold changes of 31 DE miRNAs at each time point.

Supplementary Table 3. The normalized expression values of 31 DE miRNAs.

Supplementary Table 4. Mapping rates in RNA sequencing from 18 macrophage samples.

Supplementary Table 5. The fold changes of DE genes at each time point.

Supplementary Table 6. The normalized expression values of DE genes.

Supplementary Table 7. The enriched KEGG pathways in time-series clusters

Supplementary Table 8. The robust target list of 31 DE miRNAs based on sequence complement and expression correlation.

Supplementary Table 9. The fisher test result between the targets of miRNAs and targets from public databases.

Supplementary Table 10. The top significantly enriched pathways of GSEA analysis on the targets of each miRNAs.

Supplementary Table 11. The hypergeometric test significance table between targets of DE miRNAs and DE genes at each time point.

Supplementary Table 12. The reported miRNAs involved in macrophage polarization from three independent studies.

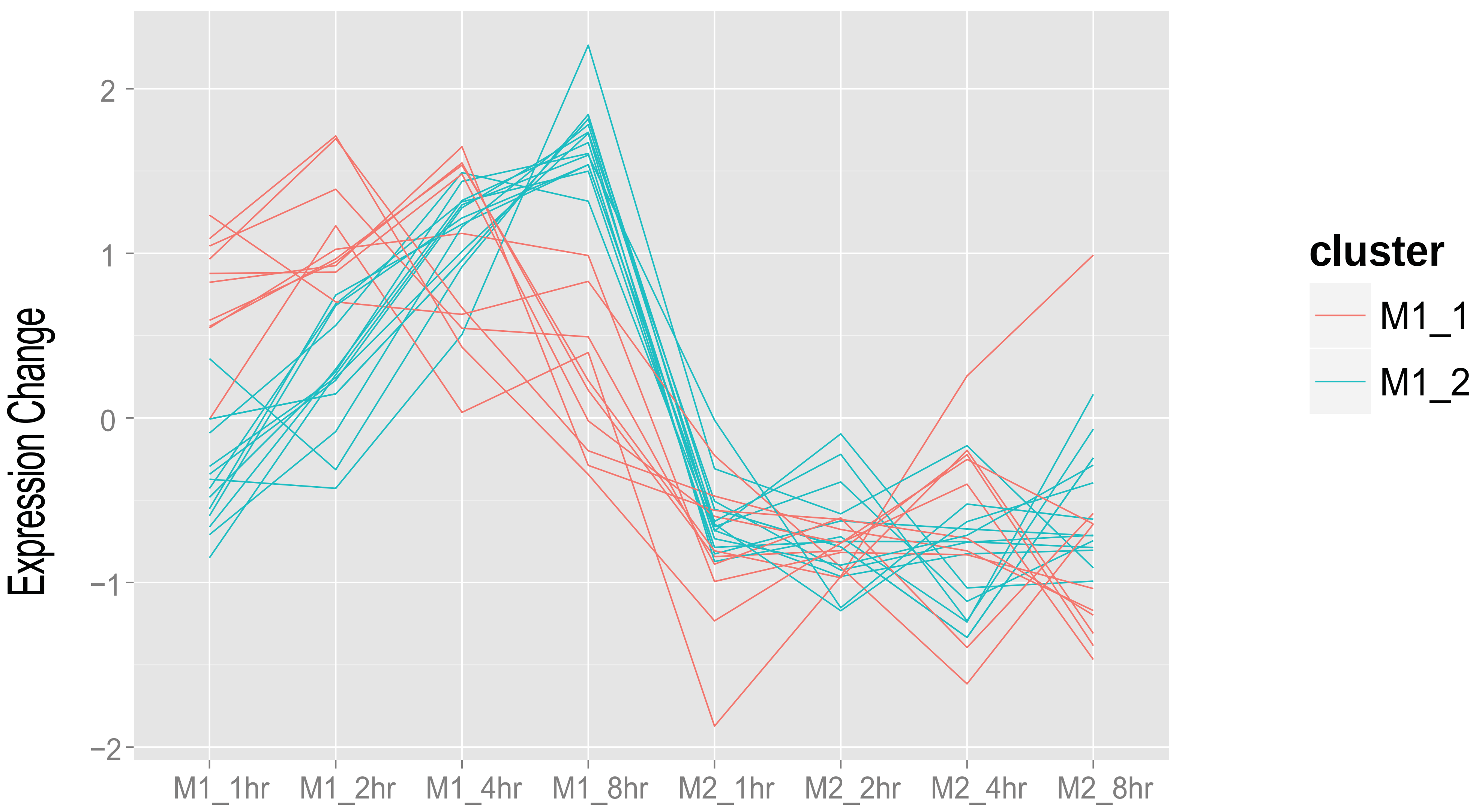

Expression Change
2
1
0
−1
−2
M1_1hr
M1_2hr
M1_4hr
M1_8hr
M2_1hr
M2_2hr
M2_4hr
M2_8hr
cluster
M1_1
M1_2

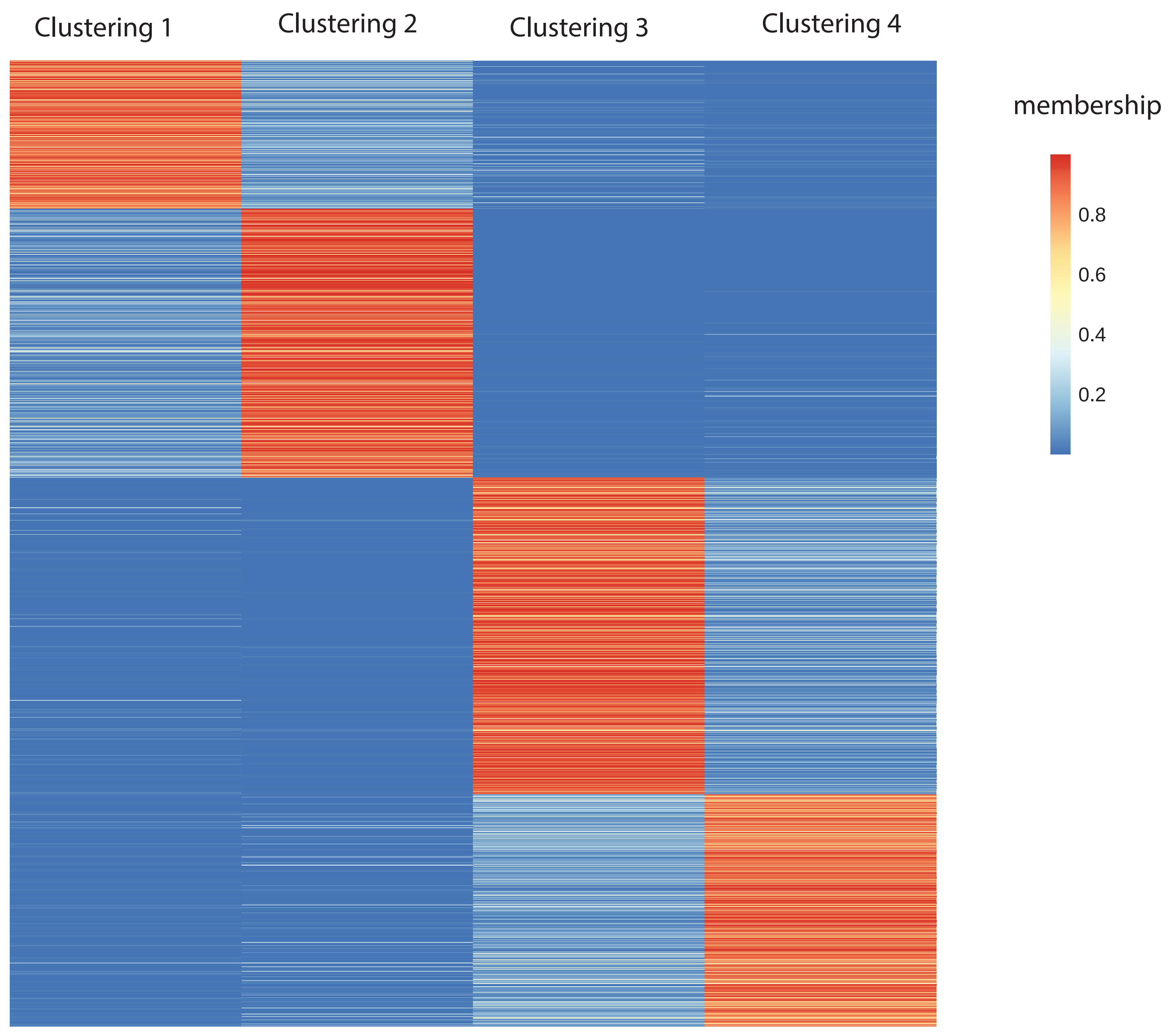
Clustering 1
Clustering 2
Clustering 3
Clustering 4
membership
0.8
0.6
0.4
0.2

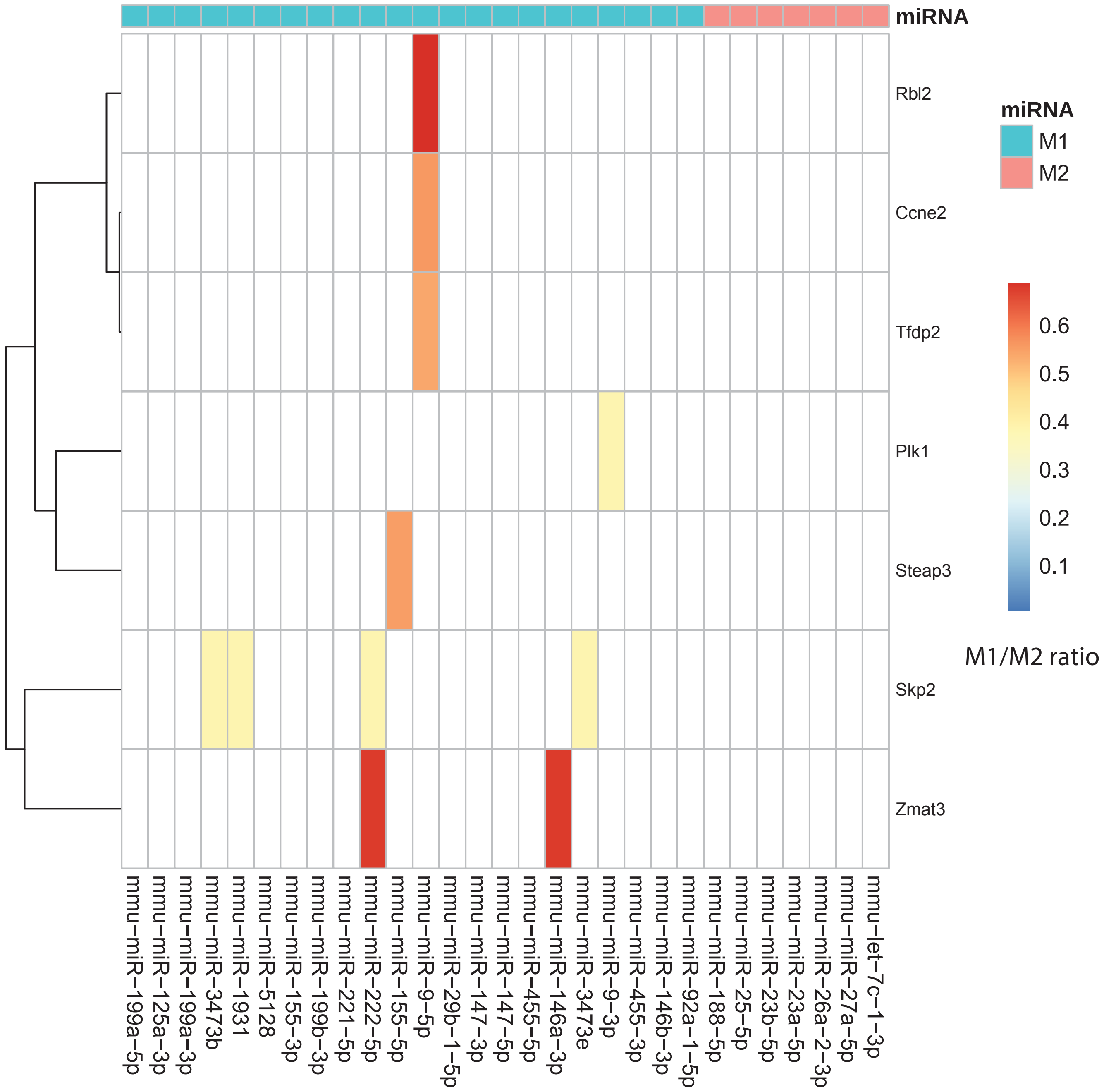

miRNA
Rbl2
Ccne2
Tfdp2
Plk1
Steap3
Skp2
Zmat3
miRNA
M1
M2
0.6
0.5
0.4
0.3
0.2
0.1
M1/M2 ratio
mmu−miR−199a−5p
mmu−miR−125a−3p
mmu−miR−199a−3p
mmu−miR−3473b
mmu−miR−1931
mmu−miR−5128
mmu−miR−155−3p
mmu−miR−199b−3p
mmu−miR−221−5p
mmu−miR−222−5p
mmu−miR−155−5p
mmu−miR−9−5p
mmu−miR−29b−1−5p
mmu−miR−147−3p
mmu−miR−147−5p
mmu−miR−455−5p
mmu−miR−146a−3p
mmu−miR−3473e
mmu−miR−9−3p
mmu−miR−455−3p
mmu−miR−146b−3p
mmu−miR−92a−1−5p
mmu−miR−188−5p
mmu−miR−25−5p
mmu−miR−23b−5p
mmu−miR−23a−5p
mmu−miR−26a−2−3p
mmu−miR−27a−5p
mmu−let−7c−1−3p

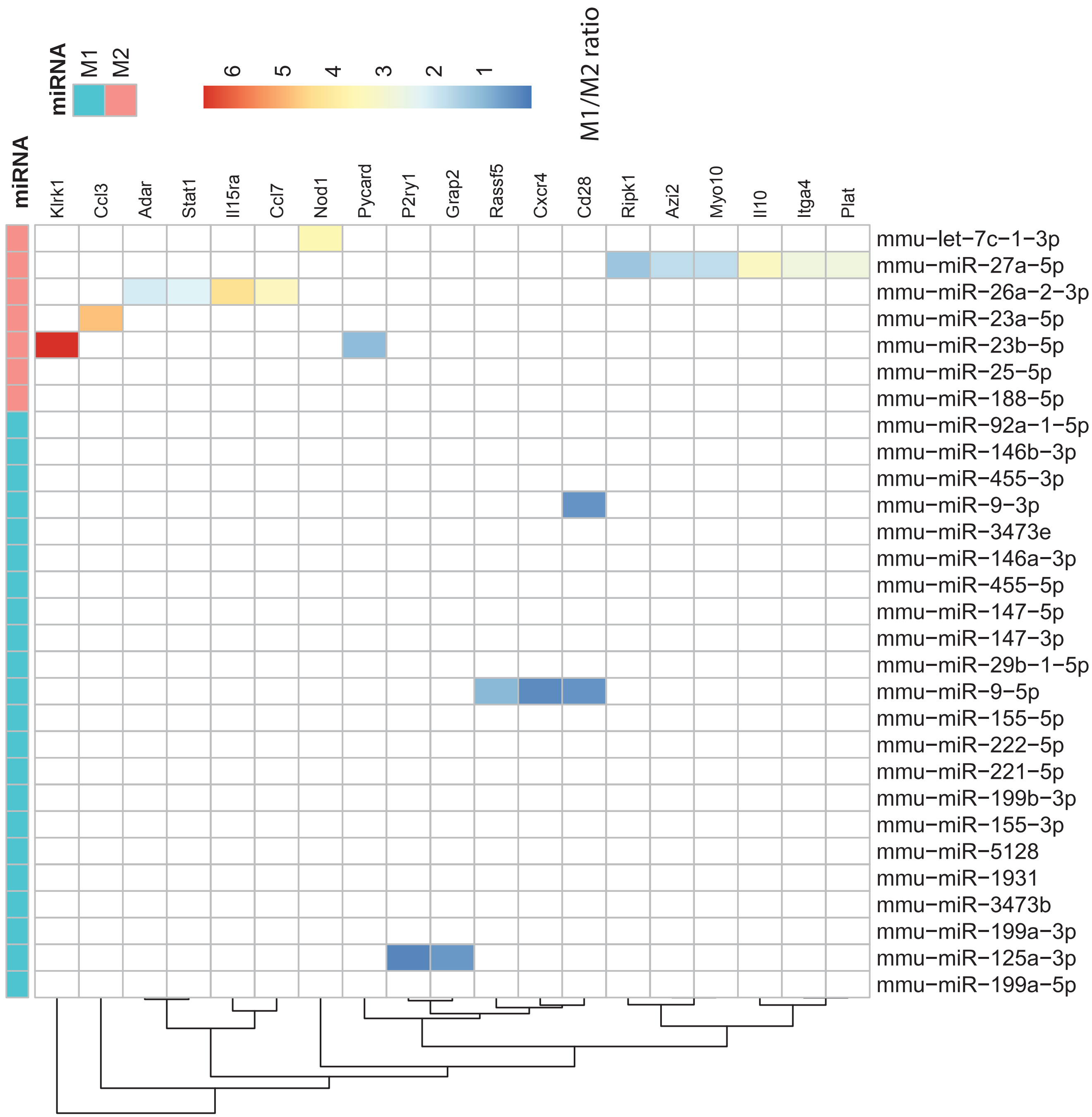
miRNA
M1
M2
6
5
4
3
2
1
M1/M2 ratio
miRNA
Klrk1
Ccl3
Adar
Stat1
Il15ra
Ccl7
Nod1
Pycard
P2ry1
Grap2
Rassf5
Cxcr4
Cd28
Ripk1
Azi2
Myo10
Il10
Itga4
Plat
mmu−let−7c−1−3p
mmu−miR−27a−5p
mmu−miR−26a−2−3p
mmu−miR−23a−5p
mmu−miR−23b−5p
mmu−miR−25−5p
mmu−miR−188−5p
mmu−miR−92a−1−5p
mmu−miR−146b−3p
mmu−miR−455−3p
mmu−miR−9−3p
mmu−miR−3473e
mmu−miR−146a−3p
mmu−miR−455−5p
mmu−miR−147−5p
mmu−miR−147−3p
mmu−miR−29b−1−5p
mmu−miR−9−5p
mmu−miR−155−5p
mmu−miR−222−5p
mmu−miR−221−5p
mmu−miR−199b−3p
mmu−miR−155−3p
mmu−miR−5128
mmu−miR−1931
mmu−miR−3473b
mmu−miR−199a−3p
mmu−miR−125a−3p
mmu−miR−199a−5p

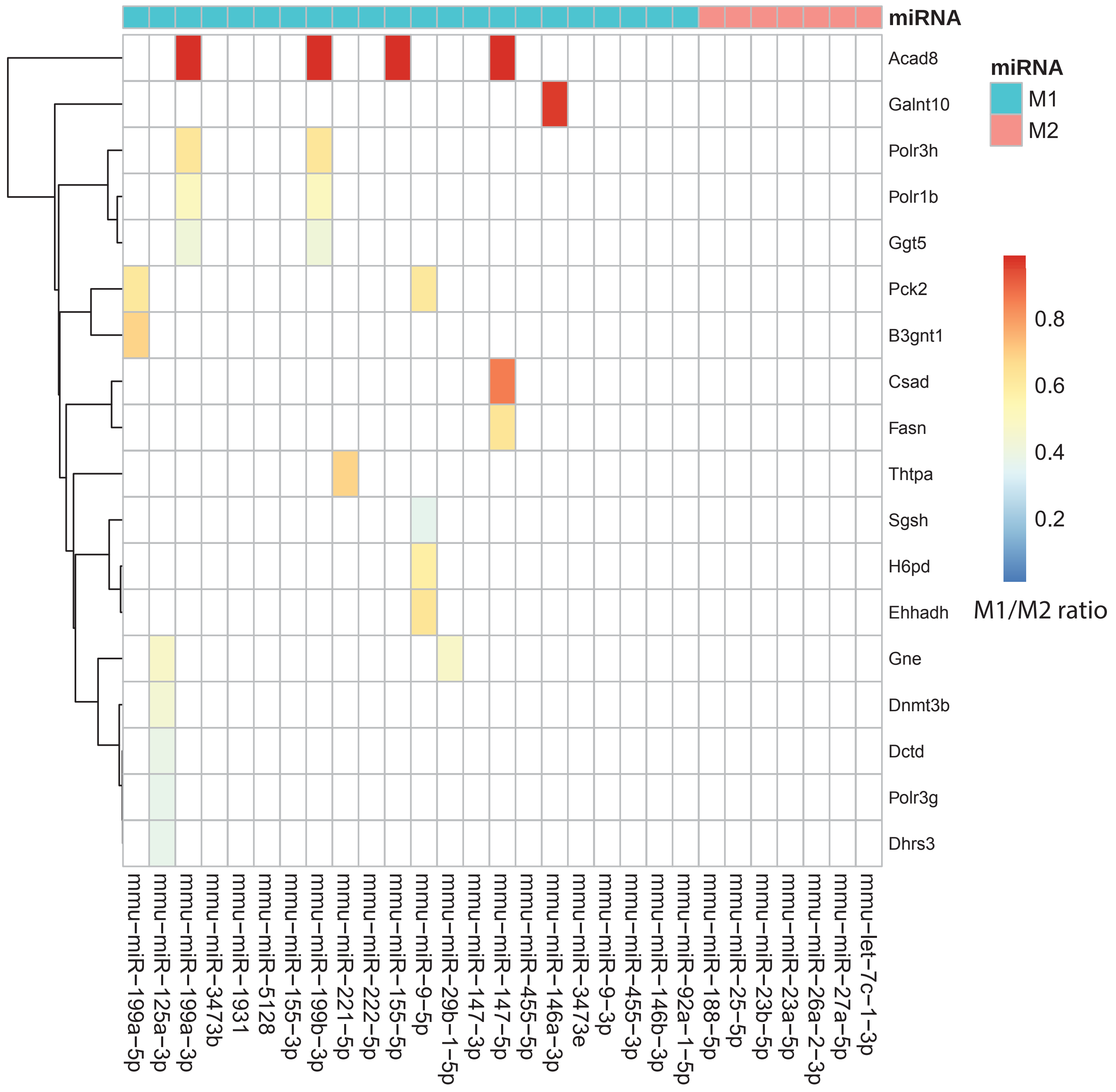

miRNA
Acad8
Galnt10
Polr3h
Polr1b
Ggt5
Pck2
B3gnt1
Csad
Fasn
Thtpa
Sgsh
H6pd
Ehhadh
Gne
Dnmt3b
Dctd
Polr3g
Dhrs3
mmu−miR−199a−5p
mmu−miR−125a−3p
mmu−miR−199a−3p
mmu−miR−3473b
mmu−miR−1931
mmu−miR−5128
mmu−miR−155−3p
mmu−miR−199b−3p
mmu−miR−221−5p
mmu−miR−222−5p
mmu−miR−155−5p
mmu−miR−9−5p
mmu−miR−29b−1−5p
mmu−miR−147−3p
mmu−miR−147−5p
mmu−miR−455−5p
mmu−miR−146a−3p
mmu−miR−3473e
mmu−miR−9−3p
mmu−miR−455−3p
mmu−miR−146b−3p
mmu−miR−92a−1−5p
mmu−miR−188−5p
mmu−miR−25−5p
mmu−miR−23b−5p
mmu−miR−23a−5p
mmu−miR−26a−2−3p
mmu−miR−27a−5p
mmu−let−7c−1−3p
miRNA
M1
M2
0.8
0.6
0.4
0.2
M1/M2 ratio
